# Denosumab combined with chemotherapy followed by anlotinib in the treatment of multiple metastases of malignant peripheral nerve sheath tumor: a case report and literature review

**DOI:** 10.3389/fonc.2024.1399021

**Published:** 2024-07-25

**Authors:** Qian Chen, Haocheng Cui, Kai Zheng, Ming Xu, Xiuchun Yu

**Affiliations:** Department of Orthopedics, The 960th Hospital of the People’s Liberation Army, Jinan, Shandong, China

**Keywords:** malignant peripheral nerve sheath tumor, anlotinib, denosumab, adjuvant chemotherapy, case report

## Abstract

Primary intraosseous malignant peripheral nerve sheath tumors (MPNSTs) are rare yet highly aggressive neoplasms originating from peripheral nerves. Typically manifesting as soft tissue masses accompanied by pain or functional impairment, these tumors pose significant challenges in management. Surgical intervention remains the cornerstone of treatment for patients with MPNST lacking distant metastasis, with generally modest success rates. In cases of recurrence and metastasis, the pursuit of effective systemic therapies has been a focus of clinical investigation. Herein, we present a case study involving an elderly female patient with refractory MPNST. In light of surgical limitations, a multimodal therapeutic approach combining chemotherapy, denosumab, and subsequent administration of anlotinib was pursued following collaborative consultation. This regimen yielded noteworthy clinical benefits, exemplifying a promising avenue in the management of challenging MPNST cases.

## Introduction

Malignant peripheral nerve sheath tumor (MPNST) represents a highly aggressive sarcoma arising from neuroectodermal cells of peripheral or cranial nerves, comprising approximately 4%–5% of all soft tissue sarcoma (STS) and contributing to an estimated 1,500 new cases annually in the European Union (1). In 2002, the WHO classification of nervous system tumors adopted MPNSTs to supplant the perplexing nomenclature encompassing malignant Schwann cell tumors, malignant schwannoma, neurosarcoma, and neurofibrosarcoma (2). With an annual incidence of one in a million and a male-to-female ratio of 2.5:1, MPNST predominately afflicts adults aged 20–50 years, with a prevalence of 10%–20% before 20 years old (3). Predominantly localized along peripheral nerves of the trunk, extremities, head and neck regions, and spine, MPNSTs exhibit histological hallmarks such as heightened cellularity, frequent mitoses, anaplasia, necrosis, infiltrative growth patterns, pleomorphism, and elevated proliferative activity (4). Metastases are identified in 40%–70% of patients, commonly affecting the lungs, liver, or bones (5). The data of Akshintala et al. have established a baseline progression-free survival (PFS) of 1.77 months in patients with recurrent or unresectable/metastatic MPNST (6).

The management of MPNST poses formidable challenges owing to its propensity for recurrence and metastasis, coupled with its limited responsiveness to systemic therapies. Complete surgical excision with wide negative margins stands as the sole curative modality for MPNST; however, its feasibility is often impeded by tumor size, location, or metastatic dissemination, leaving patients with unresectable, metastatic, or recurrent disease devoid of curative options (7). Hence, there arises an urgent imperative to delineate effective therapeutic paradigms for MPNST.

We herein present the case of a 68-year-old woman with primary MPNST devoid of neurofibromatosis-1 (NF-1). At the time of initial diagnosis, the patient presented with distant metastases, underscoring the imperative for aggressive multimodal local therapies in patients with localized MPNST for optimal disease control.

## Case report

**Figure 1:**
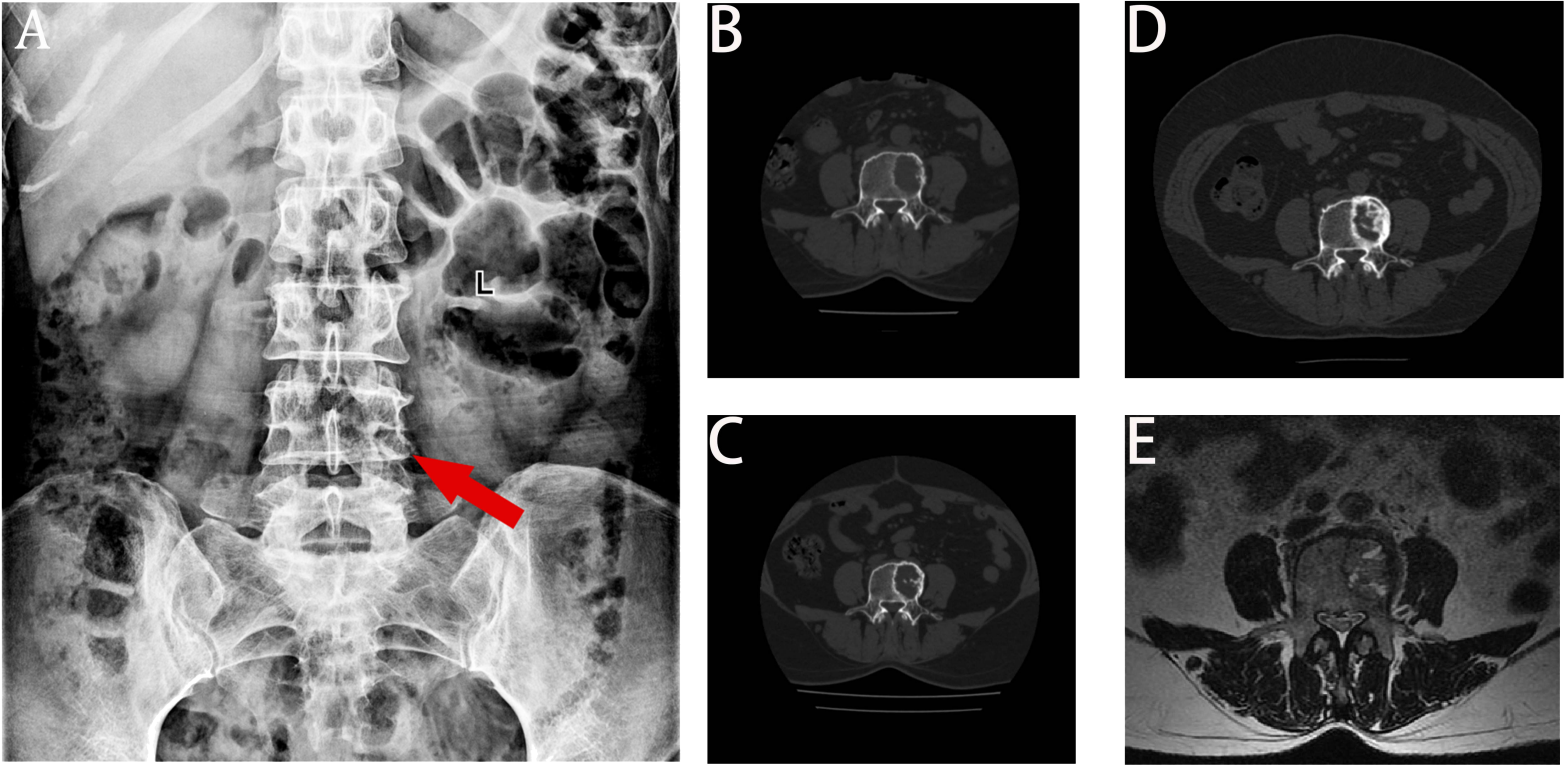
X-ray, CT, and MRI images. Panel **(A)** displays the flattening of the L4 left vertebral body height. Panel **(B)** displays the bone destruction of L4 before chemotherapy, with a moderately circular low-density shadow with a range of 3.4 cm × 2.0 cm × 2.2 cm. Panel **(C)** displays a decrease in lytic bone destruction and an increase in the cortical bone density in posterior L4 after six cycles of chemotherapy. Panel **(D)** displays obvious calcification in the bone destruction area of the L4 vertebra at the last review. Panel **(E)** displays that the left side of the L4 vertebra is slightly flattened, with patchy long T1 equal length T2 signals and high T2 pressure lipid images.

**Figure 2:**
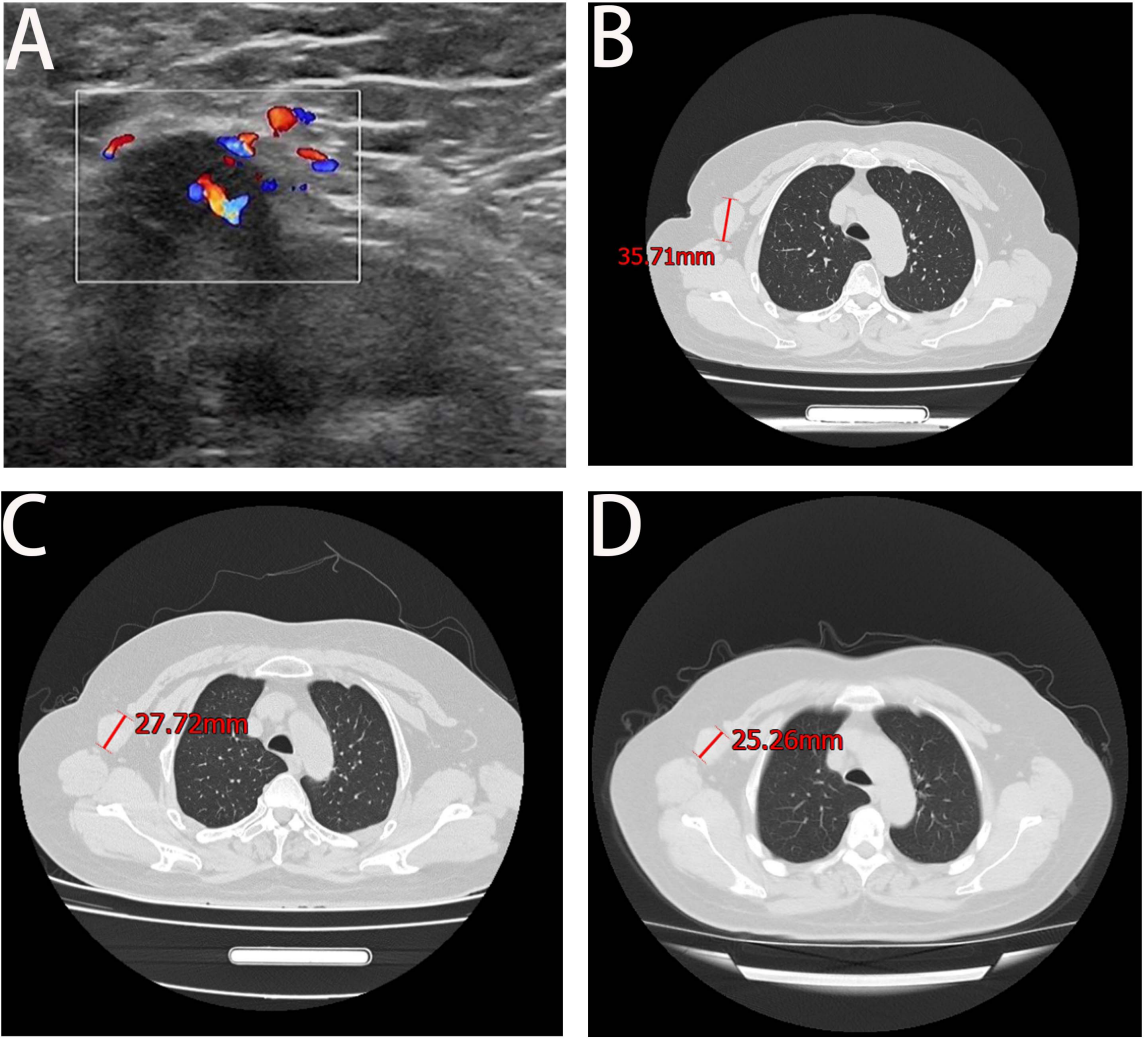
Axillary ultrasound and lung CT. Panel **(A)** shows the right axillary probe and irregularly shaped hypoechoic mass, measuring approximately 32 mm × 26 mm. Panel **(B)** shows the maximum axillary mass diameter at the time of initial admission. Panel **(C)** shows a significant reduction in the maximum diameter of the irregular soft tissue mass under the right axilla at the end of the entire chemotherapy cycle. Panel **(D)** displays the result of the February 2024 review, which indicated a decrease of approximately 2 cm in the maximum tumor diameter from the previous review.

**Figure 3:**
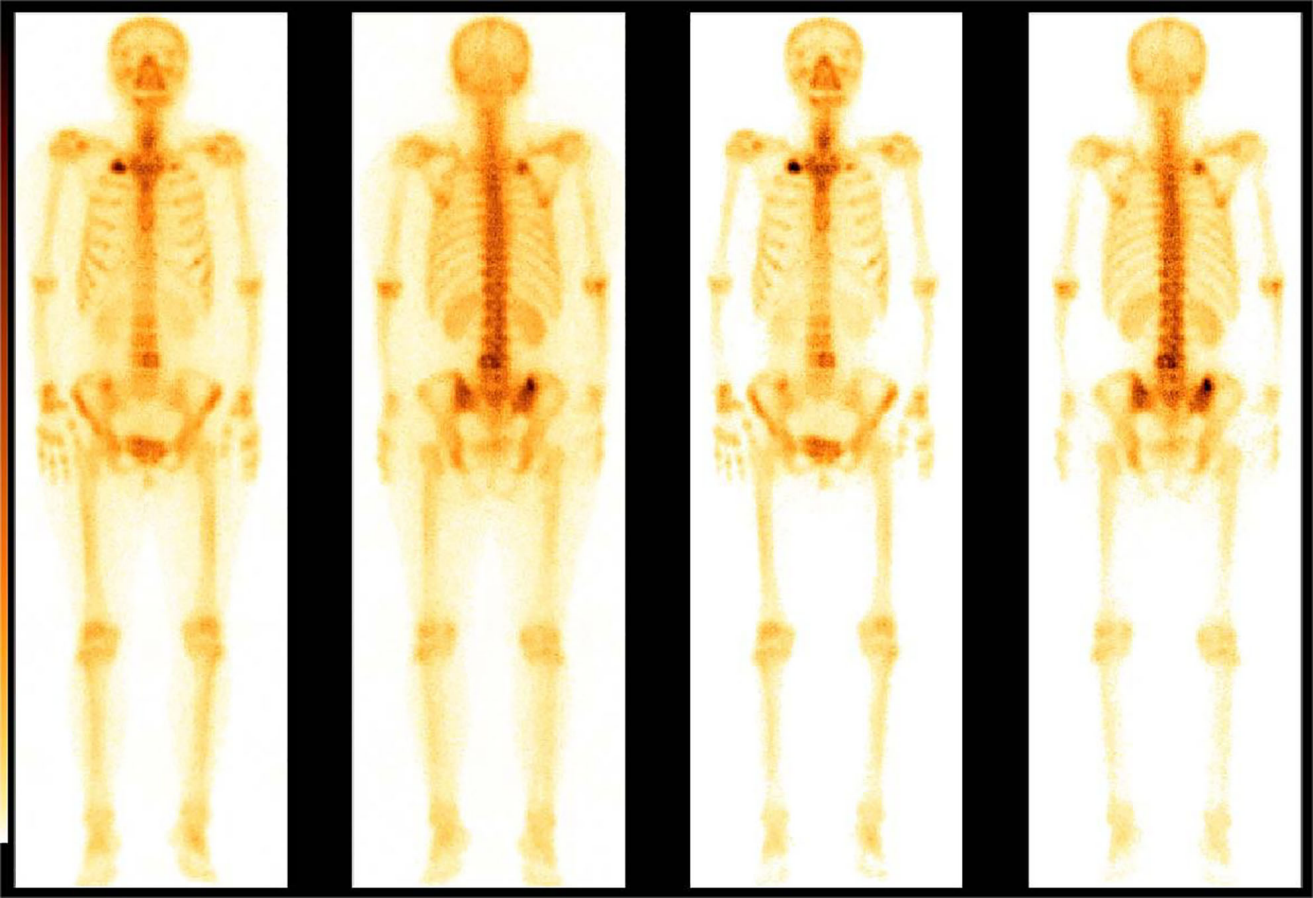
Bone scan nuclide concentration results. ECT revealed sparse radioactivity on the left side of the T4 and multiple abnormal focal nuclide concentrations on the right first rib, the L4, and the right iliac bone.

**Figure 4:**
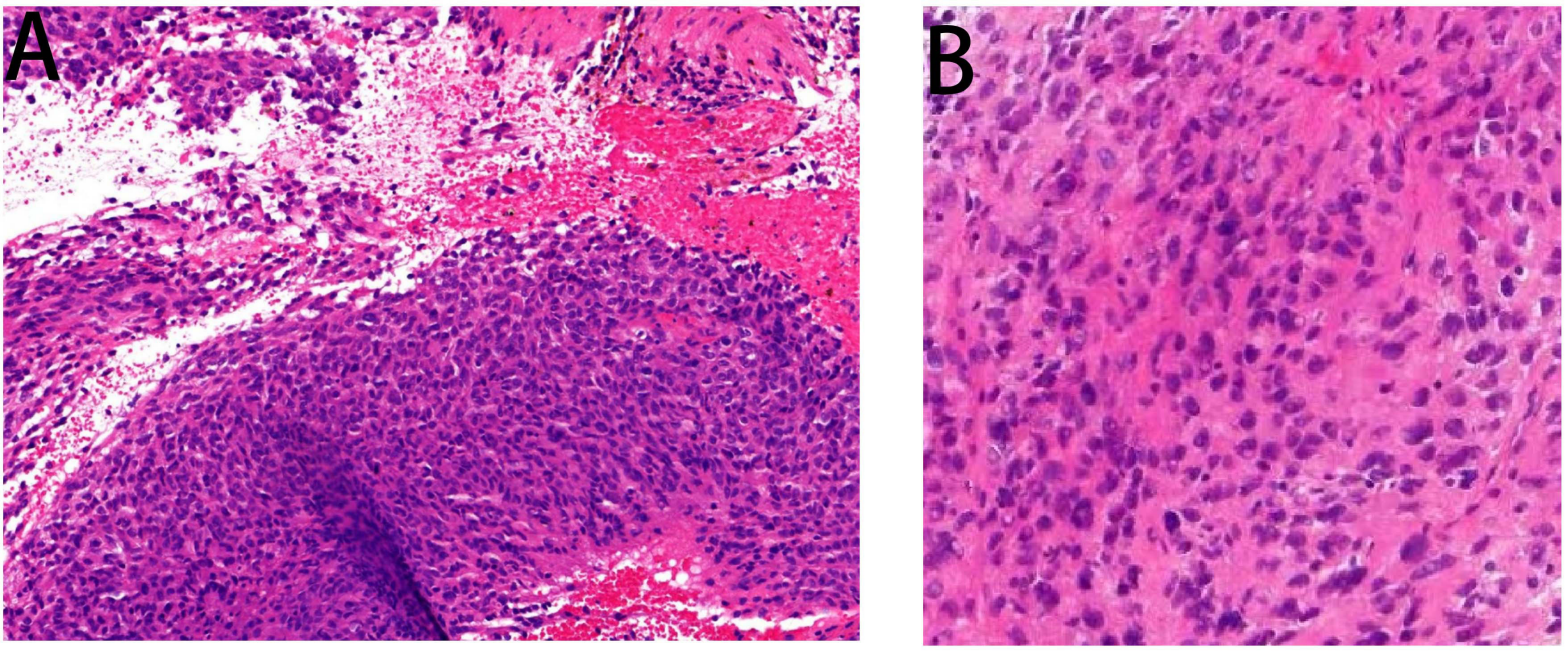
Pathology images. Panel **(A)** displays the pathological image of the right iliac bone tumor taken by a light microscope at ×200 magnification, wherein some heterotypic cell components were detected. Panel **(B)** is a pathological image of an axillary puncture captured by light microscopy under a ×200 magnifying glass. Malignant tumor cells were detected in the image.

A 68-year-old female patient presented at our hospital on 28 May 2023, with a chief complaint of “low back pain with left lower limb pain for 2 weeks.” Upon specialized physical examination, she exhibited a normal gait upon entering the ward. The patients reported left lumbar back pressure pain without radiation to the lower limb. Examination of the lumbar spine indicated normal flexion and extension activity, with left quadriceps muscle strength graded at level IV. Sensation in the lower limbs and saddle area was intact, alongside normal muscle tension. Bilateral heel–knee tendon reflexes were within normal limits, with no pathological signs detected. Normal skin temperature and color were normal in the right axilla. Palpitation revealed soft masses approximately 5 cm × 3 cm in depth, with indistinct boundaries, absence of tenderness, and immobility. Subsequent lumbar spine X-ray depicted slight flattening of the lumbar vertebra 4 (L4) vertebral body (**Figure 1A**). Computed tomographic (CT) imaging of the lumbar spine revealed flattening of the L4 vertebral body, thin and discontinuous bone cortex, and osteolytic bone destruction on the left side of the vertebra, measuring approximately 3.0 cm × 2.0 cm × 2.2 cm (**Figure 1B**). No compression of the dural sac or significant abnormalities in the surrounding soft tissue were noted. Lung CT revealed distinctive insect-like patterns of local bone destruction affecting thoracic vertebra 4 (T4) and the right first rib, with irregular soft tissue density shadows observed in the right armpit (**Figure 2B**). Lumbar magnetic resonance imaging (MRI) displayed an uneven bone damage signal in the L4 vertebral body (**Figure 1E**). Ultrasonography detected a low-echo mass measuring approximately 32 mm × 26 mm, featuring clear boundaries and an irregular shape, exhibiting uneven internal echoes and the characteristic “rat tail sign” at both ends in the right axilla (**Figure 2A**). Emission computed tomography (ECT) scans revealed multiple abnormal focal nuclide concentrations in the right first rib, L4 vertebral body, and right iliac bone, consistent with bone metastases (**Figure 3**). To determine the nature and origin of the tumors, a CT-guided puncture biopsy of the right iliac bone tumor was conducted on 29 May 2023, revealing a short spindle-cell malignant mesenchymal tumor suggestive of MPNST upon immunohistochemistry and staining (**Figure 4A**). Subsequently, on 6 June 2023, an ultrasound-guided puncture biopsy of the right axillary soft tissue tumor confirmed MPNST (**Figure 4B**). The final diagnosis was MPNST with multiple metastases.

Following thorough departmental deliberation, initiation of a chemotherapy regimen combining cisplatin with doxorubicin was decided upon. Given the predominant imaging findings of osteolytic bone destruction, denosumab, FDA-approved for preventing skeletal-related events in patients with bone metastasis from solid tumors, was adjunctively administered to inhibit osteolysis. The treatment protocol combined chemotherapy (AP regimen cisplatin (DDP) 120mg/m^2^/day ×1 day + pegylated liposomal doxorubicin (PLD) 40 mg/m^2^/day × 1 day, once every 21 days) with denosumab (120 mg, subcutaneous injection, once/4 weeks), supplemented with Vitamin D3 (600 μg daily). Throughout the treatment course, the patient experienced significant alleviation of pain symptoms compared to admission, improved sleep without medication assistance, and absence of drug-related adverse reactions. Regular imaging assessments performed before each admission facilitated the evaluation of the combination therapy’s efficacy. Following the completion of six cycles of chemotherapy, pulmonary CT scans evidenced a gradual reduction in the maximum diameter of the irregular soft tissue mass under the right axilla (**Figure 2C**), while lumbar spine CT exhibited thickening and densification of the bone cortex surrounding the L4 vertebral body, accompanied by evident bone formation and scattered calcification shadows in the area of osteolytic bone destruction (**Figure 1C**).

On 18 October 2023, the patient completed six cycles of chemotherapy combined with denosumab treatment, with lung CT indicating an absence of lung metastasis. Evaluation of the soft tissue mass in the right armpit, designated as the target lesion according to RECIST 1.1 criteria, revealed stable disease. To inhibit disease progression post-cessation of intensive chemotherapy cycles, the patient was prescribed the oral targeted drug anlotinib (12 mg/day, taken for 2 weeks followed by a 1-week break) to enhance chemotherapy sensitization and prevent disease progression. Denosumab (120 mg, subcutaneous injection, once every 4 weeks) was continued to enhance bone strength. Presently, the patient has endured the tumor for 10 months. Regular follow-up visits every 3 months for lung and lumbar CT scans were advised to monitor therapeutic efficacy and adjust the medication regimen as necessary. On 20 February 2024, a re-examination CT revealed prominent calcification in the area of bone destruction in the L4 vertebral body (**Figure 1D**), with no significant alteration in the maximum diameter of the axillary mass compared to the previous scan (**Figure 2D**).

## Discussion

Intraosseous MPNSTs represent a rare manifestation of cancer, often arising from secondary invasion from adjacent soft tissues (8). Published studies have documented only a handful of cases of primary intraosseous MPNST, with the mandible being the most common sure (approximately 50%), followed by the maxilla, spine, and, occasionally, the appendicular skeleton. In cases affecting the appendicular skeleton, intraosseous MPNSTs predominately occur in bones of the upper extremity (humerus, ulna, metacarpal, and phalanx), with involvement of the lower extremity bones being uncommon (9). The etiology and pathogenesis of primary intraosseous MPNST remain elusive. Unlike MPNST of the soft tissues, most often, primary intraosseous MPNST is not associated with NF-1. While soft tissue MPNSTs typically manifest as fusiform or eccentric masses originating from major nerves, presenting initially as painless enlarging masses, primary intraosseous MPNSTs typically present with pain, with swelling often developing later in the disease course. MPNSTs arising from major nerves commonly result in sensorimotor symptoms corresponding to the distribution of the affected nerve, with variable pain presentation (10). Results from phase II trials have indicated a median PFS of 1.77 months for relapsed refractory MPNST, with PFS rates at 2 months and 4 months being 0.42 and 0.15, respectively (6). In such cases, curative treatment is typically unattainable, and palliative systemic therapy serves as the primary approach to enhance patients’ survival and quality of life.

The challenge in cases like this lies in the complexity of achieving complete wide resection due to multiple vertebral and iliac metastases. Simple excision of the soft tissue mass under the armpit does not improve the patient’s survival and may entail the additional risk of significant functional loss. Following consultation with the patient and family, a conservative treatment approach was deemed appropriate.

Systemic treatment strategies for MPNST generally align with the general guidelines for other STS algorithms and predominantly rely on genotoxic chemotherapy, primarily serving a palliative role in the setting of metastatic diseases (11). The SARC006 phase II trial conducted by the Sarcoma Alliance for Research (SARC) suggests that NF1-associated disease predicts inferior responses to chemotherapy compared with sporadic disease (12). Although chemotherapy can mitigate tumor recurrence and distant metastasis rates, and improve patient’s quality of life of patients, it typically does not extend overall survival. While several agents have exhibited some efficacy against MPNST, including gemcitabine, docetaxel, carboplatin, etoposide, dactinomycin, cisplatinum, vincristine, cyclophosphamide, imidazole carboxamide, doxorubicin, and ifosfamide, their clinical benefits have been inconsistent (13). Until 2010, high doses of ifosfamide were commonly used; however, recent research and clinical trials have favored doxorubicin-based treatment, reflecting evolving insights into MPNST biology and affirming the superiority of such regimens (14). Notably, liposomal formulations of doxorubicin have demonstrated enhanced efficacy and reduced toxicity in MPNST xenograft models (15). Despite pathological confirmation of MPNST with multiple systemic involvement, identifying the primary lesion remains challenging. Presently, combination regimens such as anthracyclines with isocyclic phosphamide and etoposide (ICE) have emerged as a focus of clinical research (16), although their toxicity is relatively high. Considering the patient’s age, extensive bone destruction, and the primary origin of the malignant tumor from bone, we have opted for a chemotherapy regimen typically employed in osteosarcoma cases. Specifically, cisplatin has been chosen over ifosfamide due to its status as a first-line preferred treatment when combined with doxorubicin.

Upon admission, imaging studies revealed extensive osteolytic bone destruction involving multiple vertebrae and the right ilium, a condition that could not be adequately managed through chemotherapy alone and posed a risk of vertebral fractures and other adverse complications if left unchecked. Drawing from the pivotal Fracture Reduction Evaluation of Denosumab in Osteoporosis (FREEDOM) trial, which demonstrated a significant reduction in fracture risk by 68% and hip fractures by 40% over 3 years with denosumab (17), the decision was made to incorporate denosumab into the treatment regimen. Denosumab, a human monoclonal antibody that inhibits bone resorption, promotes new bone formation and delays tumor progression by binding to the receptor activator of nuclear factor-ĸB ligand (RANKL) and preventing its interaction with RANK, thus mimicking the action of osteoprotegerin (OPG) (18). Additionally, denosumab has been cleared by the FDA for use in various conditions, including osteoporosis and bone metastases (19). However, discontinuing denosumab poses challenges, as rapid reversal after discontinuation can lead to a rebound in bone turnover, potentially resulting in complications such as hypercalcemia and vertebral compression fractures (20). Therefore, a careful and deliberate strategy is essential when discontinuing denosumab (21). Limited evidence suggests that transitioning to a short course of bisphosphonate therapy with close monitoring of BMD and BTMs may mitigate bone loss and reduce multiple vertebral fracture risk (22). The medication cycle can be appropriately adjusted according to the patient’s situation, potentially extending from once every month, in the beginning, to once every 1.5 months to once every 2 months, with continued medication to prevent adverse events after withdrawal.

Anlotinib is a novel orally administered TKI targeting vascular endothelial growth factor receptor-1/2/3, PDGFR α/β, fibroblast growth factor receptor-1/2/3/4, c-Kit, and Ret (23), which demonstrates antitumor activity in patients with refractory metastatic STS, with a median PFS of 5.6 months (24). In China, anlotinib has been approved for the treatment of advanced STS based on the results of phase II and phase IIb studies (ALTER0203). Common adverse events associated with anlotinib therapy, mostly grade 1/2, include triglyceride elevation, hypertension, hand–foot skin reaction, oral mucositis, and fatigue. Zhang et al. (25) reported that switching maintenance therapy to anlotinib after chemotherapy has been significantly associated with longer median PFS and OS. Such associations may be attributed to achieving an objective response or stable disease after chemotherapy, which may select patients with good prognoses, and the delayed effects of chemotherapy may contribute to anlotinib maintenance therapy. Certain studies suggest that anlotinib with DDP significantly reduces tumor size and may reverse multidrug resistance to doxorubicin, thus enhancing chemotherapy sensitization (26). In the present case, the patient exhibited a significant reduction in axillary tumor volume at the end of the chemotherapy plus anlotinib regimen, demonstrating good short-term clinical efficacy with no serious adverse reactions. Nonetheless, further large-scale studies are warranted to confirm its effectiveness and safety.

## Conclusion

The management of MPNST remains challenging due to the lack of high-quality evidence regarding the efficacy of systemic treatments for this particular sarcoma type (27). The complexity of MPNST treatment stems not only from its high rates of local recurrence (40%–65%) and metastasis (40%–80%) but also from its poor response to conventional therapies and therapeutic options. Future research avenues may include identifying molecular markers to predict the efficacy of targeted therapy, exploring novel targeted therapy agents, investigating multi-target combination therapies, and assessing the potential synergistic effects of targeted drugs with traditional treatments. While our center has only conducted a preliminary trial of combined drug regimens, further comprehensive investigations through multi-center, large-scale studies offer the promise of uncovering effective treatments for metastatic MPNST, thereby providing renewed hope for patients with this challenging malignancy.

## Data availability statement

The original contributions presented in the study are included in the article/supplementary material. Further inquiries can be directed to the corresponding author.

## Ethics statement

The studies involving humans were approved by Department of Medical Ethics, 960th Hospital, Joint Logistic Support Force, People’s Liberation Army. The studies were conducted in accordance with the local legislation and institutional requirements. The participants provided their written informed consent to participate in this study. Written informed consent was obtained from the individual(s) for the publication of any potentially identifiable images or data included in this article. Written informed consent was obtained from the participant/patient(s) for the publication of this case report.

## Author contributions

QC: Formal analysis, Investigation, Writing – original draft. HC: Data curation, Formal analysis, Supervision, Writing – review & editing. KZ: Data curation, Formal analysis, Supervision, Writing – review & editing. MX: Data curation, Formal analysis, Supervision, Writing – review & editing. XY: Data curation, Formal analysis, Supervision, Validation, Writing – review & editing.

## References

[1] Iqbal F Jamaluddin M Bukhari F Islam OS . Malignant peripheral nerve sheath tumours in a patient with Neurofibromatosis-1. J Pak Med Assoc. (2023) 73:393–5. doi: 10.47391/JPMA.4612 36800734

[2] Bradford D Kim A . Current treatment options for Malignant peripheral nerve sheath tumors. Curr Treat Options Oncol. (2015) 16:328. doi: 10.1007/s11864-015-0328-6 25777573

[3] Somatilaka BN Sadek A McKay RM Le LQ . Malignant peripheral nerve sheath tumor: models, biology, and translation. Oncogene. (2022) 41:2405–21. doi: 10.1038/s41388-022-02290-1 PMC9035132 35393544

[4] Ortiz WJ Salazar MS Eager JJ Sajid S Cervantes M . Primary intraosseous Malignant peripheral nerve sheath tumor of the humerus: report of a rare case. Cureus. (2022) 14:e33178. doi: 10.7759/cureus.33178 36726883 PMC9886069

[5] Gupta G Maniker A . Malignant peripheral nerve sheath tumors. Neurosurg Focus. (2007) 22:E12. doi: 10.3171/foc.2007.22.6.13 17613203

[6] Akshintala S Mallory NC Lu Y Ballman KV Schuetze SM Chugh R . Outcome of patients with Malignant peripheral nerve sheath tumors enrolled on sarcoma alliance for research through collaboration (SARC) phase II trials. Oncologist. (2023) 28:453–9. doi: 10.1093/oncolo/oyac272 PMC10166173 36724001

[7] Millesi E Rechberger JS Wang H Mardini S Spinner RJ Daniels DJ . Advancements in therapeutic approaches for Malignant peripheral nerve sheath tumor. Ther Deliv. (2023) 14:385–9. doi: 10.4155/tde-2023-0014 37464750

[8] Liu W Zhang S Liu J Shao Z . Intraosseous Malignant peripheral nerve sheath tumor of 2 consecutive lumbar vertebrae: A case report and literature review. World Neurosurg. (2019) 130:459–66. doi: 10.1016/j.wneu.2019.07.117 31349078

[9] Muthusamy S Conway SA Pitcher JD Temple HT . Primary intraosseous Malignant peripheral nerve sheath tumor of the medial cuneiform: A case report and review of the literature. J Foot Ankle Surg. (2017) 56:129–34. doi: 10.1053/j.jfas.2016.05.013 27449524

[10] Kendi TK Erakar A Yildiz HY Saglik Y Erekul S . Intraosseous Malignant peripheral nerve sheath tumor with local recurrence, lung metastases and death. Skeletal Radiol. (2004) 33:223–5. doi: 10.1007/s00256-003-0678-1 14758514

[11] Karakousis CP Perez RP . Soft tissue sarcomas in adults. CA Cancer J Clin. (1994) 44:200–10. doi: 10.3322/canjclin.44.4.200 8019927

[12] Farid M Demicco EG Garcia R Ahn L Merola PR Cioffi A . Malignant peripheral nerve sheath tumors. Oncologist. (2014) 19:193–201. doi: 10.1634/theoncologist.2013-0328 24470531 PMC3926794

[13] Moretti VM Crawford EA Staddon AP Lackman RD Ogilvie CM . Early outcomes for Malignant peripheral nerve sheath tumor treated with chemotherapy. Am J Clin Oncol. (2011) 34:417–21. doi: 10.1097/COC.0b013e3181e9c08a 20838322

[14] Grimer R Judson I Peake D Seddon B . Guidelines for the management of soft tissue sarcomas. Sarcoma. (2010) 2010:506182. doi: 10.1155/2010/506182 20634933 PMC2903951

[15] Madhankumar AB Mrowczynski OD Slagle-Webb B Ravi V Bourcier AJ Payne R . Tumor targeted delivery of doxorubicin in Malignant peripheral nerve sheath tumors. PloS One. (2018) 13:e0181529. doi: 10.1371/journal.pone.0181529 29304038 PMC5755733

[16] Wang Y Katagiri H Murata H Wasa J Miyagi M Kakuda Y . Metastatic Malignant peripheral nerve sheath tumor with NF1 successfully treated with 'Gradual subtraction' ICE chemotherapy. Anticancer Res. (2020) 40:1619–24. doi: 10.21873/anticanres.14110 32132065

[17] Cummings SR San Martin J McClung MR Siris ES Eastell R Reid IR . Denosumab for prevention of fractures in postmenopausal women with osteoporosis. N Engl J Med. (2009) 361:756–65. doi: 10.1056/NEJMx090058 19671655

[18] Boyce AM . Denosumab: an emerging therapy in pediatric bone disorders. Curr Osteoporos Rep. (2017) 15:283–92. doi: 10.1007/s11914-017-0380-1 PMC5554707 28643220

[19] Visgauss JD Lazarides A Dickson B Cardona D Sheth M DeWitt SB . Treatment of chondroblastoma with denosumab: A case report with a correlative analysis of effect on the RANK signaling pathway. JBJS Case Connect. (2021) 11:e20.00178. doi: 10.2106/JBJS.CC.20.00178 33999872

[20] Pan KS Boyce AM . Denosumab treatment for giant cell tumors, aneurysmal bone cysts, and fibrous dysplasia-risks and benefits. Curr Osteoporos Rep. (2021) 19:141–50. doi: 10.1007/s11914-021-00657- PMC9533232 33616817

[21] Tay WL Tay D . Discontinuing denosumab: can it be done safely? A review of the literature. Endocrinol Metab (Seoul). (2022) 37:183–94. doi: 10.3803/EnM.2021.1369 PMC9081316 35417954

[22] Sølling AS Tsourdi E Harsløf T Langdahl BL . Denosumab discontinuation. Curr Osteoporos Rep. (2023) 21:95–103. doi: 10.1007/s11914-022-00771-6 36564572

[23] Liu J Deng YT Jiang Y . Switch maintenance therapy with anlotinib after chemotherapy in unresectable or metastatic soft tissue sarcoma: a single-center retrospective study. Invest New Drugs. (2021) 39:330–6. doi: 10.1007/s10637-020-01015-z 32974853

[24] Chi Y Fang Z Hong X Yao Y Sun P Wang G . Safety and efficacy of anlotinib, a multikinase angiogenesis inhibitor, in patients with refractory metastatic soft-tissue sarcoma. Clin Cancer Res. (2018) 24:5233–8. doi: 10.1158/1078-0432.CCR-17-3766 29895706

[25] Zhang RS Liu J Deng YT Wu X Jiang Y . The real-world clinical outcomes and treatment patterns of patients with unresectable locally advanced or metastatic soft tissue sarcoma treated with anlotinib in the post-ALTER0203 trial era. Cancer Med. (2022) 11:2271–83. doi: 10.1002/cam4.4613 PMC9160813 35191609

[26] Cai M Zhu J Zhou G . Efficacy and safety of treating refractory bone and soft tissue sarcoma with anlotinib in different treatment patterns. Comput Math Methods Med. (2022) 2022:3287961. doi: 10.1155/2022/3287961 35991143 PMC9388280

[27] Sobczuk P Teterycz P Czarnecka AM Świtaj T Koseła-Paterczyk H Kozak K . Systemic treatment for advanced and metastatic Malignant peripheral nerve sheath tumors-A sarcoma reference center experience. J Clin Med. (2020) 9(10):3157. doi: 10.3390/jcm9103157 33003503 PMC7601777

